# Prognostic Factors of Biologic Therapy in Pediatric IBD

**DOI:** 10.3390/children9101558

**Published:** 2022-10-14

**Authors:** Anna Buczyńska, Urszula Grzybowska-Chlebowczyk

**Affiliations:** Department of Pediatrics, Medical University of Silesia, 40-055 Katowice, Poland

**Keywords:** pediatric IBD, predictors of poor outcome, infliximab

## Abstract

This was a retrospective cohort study aimed at identifying parameters measured at diagnosis of pediatric IBD to predict subsequent biologic therapy, as an equivalent to an unfavorable clinical course. Identification of predictors of poor outcomes is an important issue in current ECCO guidelines on pIBD. The study population consisted of 119 children with Crohn’s disease and 112 with ulcerative colitis, diagnosed and monitored for at least 1 year from 2009–2019. The population was divided into the study groups separately: 39 children with CD and 14 with UC who received biologics before the age of 18 y compared to 80 with CD and 98 with UC who did not. The combined analysis of 53 biologic therapy recipients vs. 178 non-recipients with IBD was also conducted. Logistic regression tests (OR, RR) and sensitivity, specificity, PPV, and NPV were used. Factors significantly correlated with subsequent biologic therapy were perianal disease, complicated disease behavior, high PCDAI (CD), fatigue, hypoalbuminemia, high PUCAI (UC) and fever, fatigue, hypoalbuminemia, hypoproteinemia, and elevated CRP (IBD). Marginally significant factors were ileocecal disease, elevated serum IgA, anemia, and L4a–L4b coexistence. Apart from parameters already accepted as POPO (B2/3, perianal disease), interesting observations are the significance of IgA, L4a–L4b in CD, and hypoalbuminemia in UC.

## 1. Introduction

Inflammatory bowel disease (IBD) and its main types, i.e., Crohn’s disease and ulcerative colitis, most commonly present in young adults; however, IBD also develops in children and older adults. Therefore, based on the age at initial diagnosis, IBD can be categorized into pediatric-onset IBD and its subcategories (early-onset, very early-onset, and infantile IBD), adult-onset and late-onset IBD [1,2,3]. The pathogenesis of IBD is complex; genetic susceptibility associated with single nucleotide polymorphism (SNP) intertwines with environmental influences (diet, factors affecting gut microbiota). The latter modify the intraepithelial cell response, autophagy mechanisms, and proinflammatory pathways, including IL12, IFN-γ, and TNFα [4,5,6]. Presently, the most advanced commonly used in clinical practice treatment for IBD is biologic therapy targeting cytokine signaling pathways; the first biological response modifier was infliximab, an anti-TNFα antibody [6]. Currently, several anti-TNFα and anti-integrin agents as well as anti-IL 12-23 are available [7].

Determination of a patient’s eligibility for biologic therapy was initially based on the so-called step-up strategy, i.e., progressive intensification of treatment depending on clinical symptoms and individual response to standard PIBD care [8]. Parallel observations were made to establish whether a top-down strategy could be more beneficial for at least some patients [9]. Undoubtedly, both strategies have benefits and concerns; the underlying idea is to lower the risk for systemic (extraintestinal) and local (strictures, fistulas, segmental resection) IBD complications. Adverse effects of chronic medication are also of importance.

The 2014 consensus guidelines of ESPGHAN/ECCO described the predictors of poor outcome to be considered while selecting the treatment [10]. The Guideline Update of 2020 presented a table with risk factors based on the Paris classification parameters at diagnosis, additional risk factors, and resultant low, medium, and high-risk stratification with suggested induction therapy [11]. In light of these guidelines, patient stratification became a fact, facilitating the identification of patients who could potentially benefit from the top-down approach. Nevertheless, the guidelines also indicated that different researchers specified different risk factors. Furthermore, the research underlying the ECCO Guidelines on Management in Crohn’s disease mainly focused on adult patients; prediction of poor outcomes was therefore extrapolated from the adult to pediatric populations. Yet it should be remembered that children with IBD might suffer complications that do not occur in adult patients, i.e., linear growth impairment, pubertal delay, and other effects of chronic disease on adolescent development [12,13]. Consequently, identification of potential risk factors and more detailed reports on IBD course in the abovementioned pediatric subcategories remain essential for clinical practice.

The aim of the study was to evaluate the clinical features and laboratory results indicating the subsequent need for biologic therapy in children and adolescents.

## 2. Material and Methods

### 2.1. Study and Control Group Definition

We retrospectively analyzed the medical records of 231 children with inflammatory bowel disease who had been diagnosed in the Department of Gastroenterology, Upper Silesian Child Health Centre, the Medical University of Silesia since the launch of the biologic IBD therapy program in the Centre (i.e., 2009 for Crohn’s disease and 2012 for ulcerative colitis) until the end of 2019. The follow-up period continued up to the age of 18 years when patients are transferred from pediatric to adult care, and only patients who were monitored for at least one calendar year following their diagnosis were enrolled in the study. IBD was diagnosed based on the original Porto criteria (2005) [14] and revised Porto criteria (2014) [15]. The total study population consisted of 119 patients with Crohn’s disease and 112 patients with ulcerative colitis. The study group was then selected, comprising 39 children with Crohn’s disease and 14 with ulcerative colitis who ultimately received biologic therapy. Those who did not, i.e., 80 children with Crohn’s disease and 98 with ulcerative colitis, formed the control group. Separate analyses were performed for Crohn’s disease (39 vs. 80 patients), ulcerative colitis (14 vs. 98 patients), and for the whole IBD population (53 biologic therapy recipients and 178 non-recipients). The exclusion criterion was a monitoring time of less than 1 year. Hence, all teenagers diagnosed with IBD over the age of 17 were excluded and the study group only comprised pediatric-onset IBD classified as A1a and A1b according to the Paris classification [1]. Fifty children of the study group were initially treated with infliximab; four non-responders were switched to adalimumab. In three children the therapy was started with adalimumab. As qualification criteria for biological treatment are common for both drugs, they were all analyzed in one study group.

Clinical and laboratory parameters at the time of IBD diagnosis were analyzed to identify risk factors for unfavorable clinical courses resulting in the subsequent adoption of biologic therapy. The following parameters were analyzed based on the patients’ medical records.

### 2.2. Patients’ History, Symptoms, Growth, and Nutritional Status

- Disease symptoms at diagnosis;
- Family history of IBD (first-degree relatives);
- Anthropometric measurements for nutritional status assessment based on percentile charts of the Polish OLAF study for children 7–18 years old [16] or WHO child growth standards in younger children [17]. Children were considered underweight if they met any of the following criteria: weight below the third percentile, weight percentile two channels lower than the height percentile, and BMI equivalent to the underweight percentile of the OLAF chart. Short stature was diagnosed in children below the third percentile for height and those with growth impairment as defined in the Paris classification [1];
- Disease activity at diagnosis using the PCDAI and PUCAI scores [18,19].

### 2.3. Additional Tests Results

In this retrospective cohort study laboratory parameters from patients’ first hospitalizations were extracted from their medical histories. During routine diagnostic tests performed at the Upper Silesian Child Health Centre, complete blood count was evaluated using fluorescein flow cytometry, and ASCA and *p*-ANCA status was assessed by indirect immunofluorescence technique. Serum albumin level and serum total protein level were determined using quantitative colorimetry. CRP, IgA, IgG, and IgE concentrations were measured by immunoturbidimetry.

For further analysis, laboratory parameters were analyzed qualitatively—the tested parameters could be within the normal range for age or elevated/decreased—depending on the parameter, as detailed in the results section.

Upper GI endoscopy and colonoscopy results were analyzed, from which both macroscopic and histological data were extracted, and together with MRI enterography the results served to assess disease location and behavior (when applicable) of each patient, as defined in the Paris classification. Therefore, Crohn’s disease patients were classified as having L1-distal 1/3 of ileum or limited ileocecal disease, L2-Crohn’s colitis, L3-ileocolonic disease, L4a,b-upper GI involvement either proximal or distal to Treitz’s ligament. The Paris classification states that L4 can coexist with L1-3. UC patients could present with disease extents classified as E1-proctitis, E2-disease distal to the splenic flexure, E3-disease distal to the hepatic flexure, and E4-pancolitis. Crohn’s disease behavior presentations were classified as B1—uncomplicated, inflammatory, B2 stricturing, B3 penetrating, and B2B3—both stricturing and penetrating [1].

Based on histological examination, the presence of eosinophilic infiltrates in GI mucosa was also assessed as a potential prognostic factor.

### 2.4. Endpoint Definition

Adoption of biologics was chosen as the primary clinical endpoint indicating disease severity and patient’s resistance to standard PIBD care. All patients who were found eligible for biologic therapy had met the criteria of severe exacerbation of IBD and steroid dependence or steroid resistance. The group also comprised patients presenting with persistent perianal disease, which is a sole and independent criterion of patient inclusion in anti-TNF treatment program financed by the Polish National Health Fund, but all of those patients concomitantly fulfilled the criterion of severe exacerbation of the disease.

### 2.5. Statistical Analysis

The normality of data distribution was analyzed with the Shapiro–Wilk test. Activity indices in the study (biology +) and control (biology −) groups were analyzed with ANOVA. The Mann–Whitney U test was used to compare patients’ age at IBD onset. The null hypothesis of the equal group size distribution regarding disease symptoms was tested with the chi-square test of independence or the Fisher exact test when appropriate. Risk factors for biologic therapy were determined using logistic regression models. The sensitivity and specificity of selected parameters were calculated as factors predictive of the ultimate need for biologic therapy; positive and negative predictive values thereof were also estimated. All parameters were computed with 95% confidence intervals (CI). The correlation strength of categorical non-dichotomous variables was determined with Spearman’s rank correlation coefficient and Cramer’s V. For dichotomous variables, Yule’s coefficient and adjusted C-Pearson’s correlation coefficient were calculated.

## 3. Results

The youngest children were an 11-month-old girl with Crohn’s disease and a 36-month-old boy with ulcerative colitis. The mean time interval between diagnosis and biologic therapy was 60 weeks for CD and 82 weeks for UC (the latter decreased to 54 weeks after the exclusion of a child qualified for biologic treatment at 9 years of UC diagnosis). One patient with CD was qualified for biologic treatment during his first (diagnostic) hospital admission.

### 3.1. Crohn’s Disease

Boys accounted for 60.5% of the Crohn’s disease subpopulation but the predominance was less pronounced in the biologic therapy group (51.3%). However, female gender did not prove a significant risk factor for starting biologic therapy (OR 1.76; 0.81–3.84; *p* = 0.15). Family history was positive for IBD in 7.4% and 10% of children with or without biologic therapy, respectively, and this parameter was not related to the subsequent adoption of biologics (OR 0.75; 0.21–2.71; *p* > 0.99).

#### 3.1.1. Crohn’s Disease—Paris Classification Parameters

Regarding the patients’ age, patients falling within the A1a and A1b age categories of the Paris classification were included. The odds ratio of biologic treatment prior to reaching 18 years of age did not differ significantly between these age subgroups (A1a vs. A1b: OR = 1.9397; 0.62–6.08; *p* = 0.2555). However, the mean age at diagnosis expressed in full months was significantly lower in biologic therapy recipients compared to non-recipients (151 months vs. 161.8, i.e., 12.5 years vs. 13.5 years; *p* = 0.0168). The proportion of children presenting with the defined Paris parameters is displayed in the Table 1.

Biologics proved necessary in 40.63% of CD children who presented with ileal disease (L1) and 23.6% of patients with other disease locations (L2 or L3). L1 turned out to be a borderline risk factor for subsequent biologics; L1 vs. L2/L3: OR 2.21; 1.004–4.97; *p* = 0.0534; RR = 1.719 (1.002–3.04). L1 allowed to predict the need for biologic therapy with a sensitivity of 0.6667 (CI 0.49–0.81) and specificity of 0.525 (0.41–0.64). Other disease locations at diagnosis had a negative predictive value for biologics, equaling to 76% (NPV = 0.7636, 0.63–0.87).

To verify a hypothesis that the involvement of the terminal ileum as such could be of significance, with or without colonic involvement, children with L1/L3 were compared to patients with Crohn’s colitis (L2). No correlation was revealed: 34% of children with L1/L3 and 26% of those with L2 received biologic therapy (OR 1.44, 0.48–3.87, *p* = 0.6019).

Regarding disease behavior, no patients presented with B2B3, and one child had penetrating disease (B3). Hence, inflammatory type (B1) was compared to complicated disease types together (B2 or B3). A strong correlation was revealed between complicated disease behavior at diagnosis and subsequent initiation of biologic therapy; OR = 2.86; 1.28–6.54, *p* = 0.0169, sensitivity of 0.4358 (0.2781–0.6037) and specificity of 0.7875 (0.6817–0.871); the PPV and NPV were 50% (32.4–67.5%) and 74.1% (63.47–83.01%), respectively. Correlation strength was determined with Spearman’s rank correlation coefficient R = 0.1872, *p* = 0.0415.

Upper gastrointestinal tract involvement was also evaluated (locations L4a and L4b). In a separate analysis, it did not increase the risk for subsequent adoption of biologic therapy. However, in the case of involvement of GI segments proximal and distal to the ligament of Treitz in the same patient (L4aL4b concomitance), the PPV for biologic therapy was 66% (22.22–95.6%) while the NPV was 69% (59.6–77.38%). A correlation between L4aL4b and the risk for biologics did not reach the level of statistical significance, although a tendency toward such correlation was noted (OR 4.46; 0.99–23.95; *p* = 0.0891).

Perianal disease strongly increased the probability of subsequent adoption of biologic therapy (OR 3.7; 1.2–11.68; *p* = 0.0356). Growth impairment/severe growth impairment was not related to subsequent use of biologics (the respective ORs were 1.35; 0.57–3.22 and 2.17; 0.59–7.76).

Table 2 presents a summary of statistical analyses of the Paris classification parameters as predictors of biologics adoption in patients below the age of 18.

#### 3.1.2. Crohn’s Disease Activity

**Figure 1:**
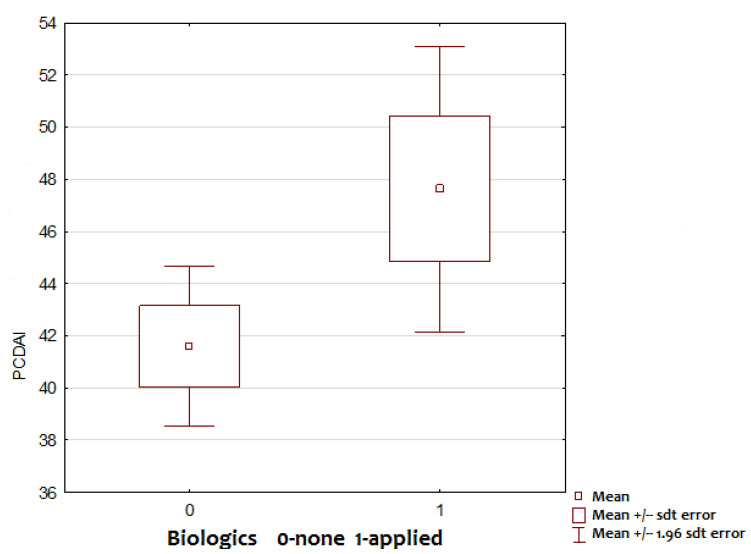
PCDAI.

Children who were ultimately treated with biologics had higher PCDAI scores at diagnosis (47.63 vs. 41.59; *p* = 0.0441; Figure 1). Nevertheless, the mean PCDAI scores indicated severe disease activity in both groups.

Patient distribution in the categories of remission–mild/moderate/severe disease activities at diagnosis differed significantly between the study and control groups. Nearly eighteen percent (17.9%) of the 39 future biologic therapy recipients at the diagnosis of CD presented with PCDAI qualified within the remission–mild range, while 10.3% presented with moderate, and 71.8% with severe disease activity. In the control group of 80 biologics non-recipients this was 8.9%, 30%, and 61.1%, respectively (chi^2 8.14, *p* = 0.04317; Rho = 0.0493).

#### 3.1.3. Crohn’s Disease—Clinical Manifestations

Disease symptoms due to which the children had been referred for diagnostic tests were analyzed; symptom distribution was compared between the recipients and non-recipients of biologic therapy in the Table 3. Analysis revealed that fever occurred more frequently in those study participants who subsequently received anti-TNF-α biologics with borderline significance (*p* = 0.078). It should also be emphasized that three out of four children with aphthous stomatitis needed biologic therapy. Hence high specificity of this parameter for the prediction of biologic therapy; however, the parameter’s sensitivity is low (only 4 out of 119 children presented with this symptom). The OR of aphthous stomatitis is relatively high with a right shifted confidence interval (>1) but the *p*-value higher than 0.05 and therefore does not allow making predictions beyond the study population.

#### 3.1.4. Crohn’s Disease—Laboratory Tests Results

An increase in IgA to age-related normal levels and moderate/severe anemia (WHO classification) turned out to be predictive of subsequent biologic therapy. Hypoproteinemia at diagnosis was another risk factor; however, this parameter had extremely low sensitivity due to a low number of children with protein deficiency (*n* = 3, all received biologic therapy). Anti-Saccharomyces cerevisiae antibodies (ASCAs) were present in approximately 40% of biologic therapy recipients and half of the non-recipients. Double positivity was diagnosed in around 35% of the children in both groups. ASCA were not a risk factor of subsequent biologics; OR and RR of ASCA positivity for IgA or IgG were < 1. The OR and RR values for double positivity were > 1 but statistically insignificant.

IgE elevation, IgE-dependent class 2 food allergy diagnosed for any food allergen, and eosinophilic infiltrates in GI mucosa were also assessed in both study and control groups; no relationship was revealed between these features and subsequent use of biologic therapy. All assessed laboratory test results for Crohn’s disease are presented in Table 4.

**Figure 2:**
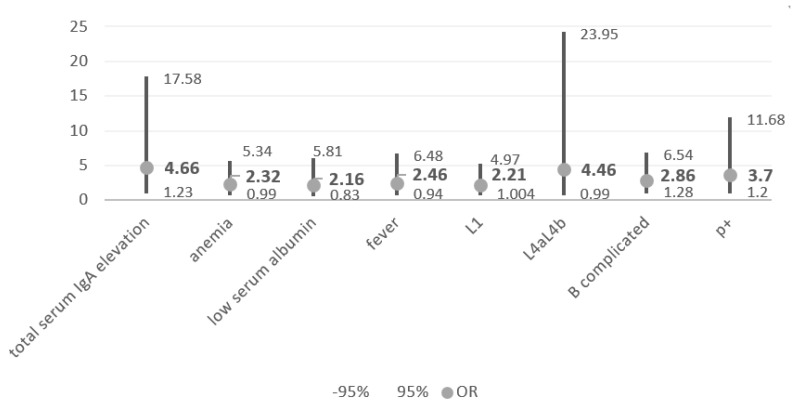
CD—risk factors [OR].

Study parameters that turned out to be borderline or significant predictors of future biologic therapy in patients with Crohn’s disease are summarized in Figure 2.

### 3.2. Ulcerative Colitis

#### 3.2.1. Ulcerative Colitis—Paris Classification Parameters and Disease Activity

Ulcerative colitis (UC) was diagnosed at a younger age in children who subsequently received biologic therapy than in those who did not (mean age 10.5 vs. 12 years, *p* = 0.14156) in the Table 5; the difference was not statistically significant.

Disease extent (E) was not related to subsequent adoption of biologic therapy. Pancolitis was the most frequent UC form both among biologic therapy recipients and non-recipients. A total of 8 (17.4%) of 46 children with pancolitis (E4) received biologics while only six (9%) of the remaining 66 UC sufferers (E1–E3) had this treatment.

Backwash ileitis and macroscopic rectal sparing (MRS) were not risk factors for biologics (no biologic therapy recipients and only three biologic therapy non-recipient children had MRS; OR = 0.94; OR 0.05–19.16; *p* = 0.97).

**Figure 3:**
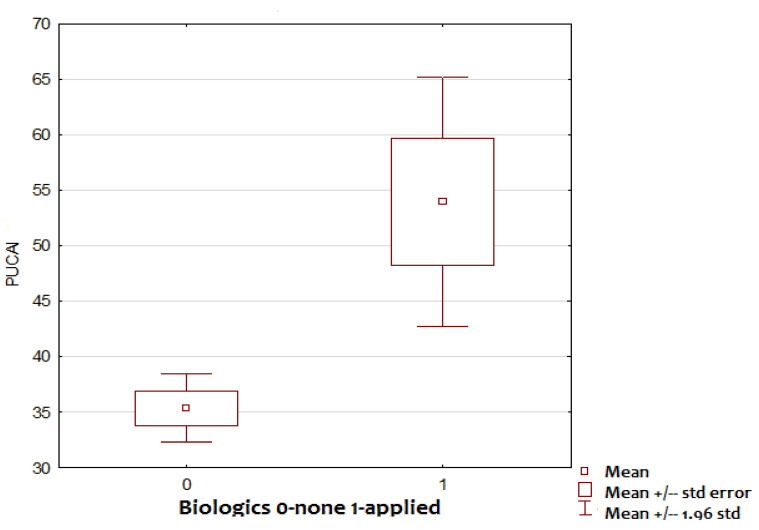
PUCAI.

The mean PUCAI scores at diagnosis were 53.9 and 35.4 in biologic therapy recipients and non-recipients, respectively; the difference was statistically significant (*p* = 0.000114; Figure 3). More than half (53.84%) of 13 children with PUCAI scores indicating severe disease received biologics, compared to only 7 (7%) out of 99 mild-moderate UC participants. The risk of demanding biologics for PUCAI at diagnosis ≥ 65 compared to mild-moderate disease was as follows: OR = 14.61 (CI 3.27–70.84); RR = 7.62 (3.18–18.24); *p* < 0.001; Yule’s Q = 0.8776; *p* < 0.00001; sensitivity = 0.5 (0.2304–0.7696). specificity = 0.9388 (0.8714–0.9772). PPV 0.5385 (0.314–0.7493). NPV = 0.9293 (0.8097–0.9367).

#### 3.2.2. Ulcerative Colitis—Demographic Data, Symptoms, and Additional Tests Results

Contrary to Crohn’s disease, the majority of UC patients were girls (60.7%); they also accounted for 64.3% of biologic therapy patients. However, female gender did not prove a statistically significant risk factor: OR = 1.19; 0.37–3.82; *p* = 0.77. Neither did positive family history, which was only noted in four non-recipients, OR = 0.72; 0.04–14.17; *p* = 0.8315.

Fatigue rates at UC diagnosis differed significantly between the biologic therapy recipients and non-recipients and strongly correlated with subsequent biologics. The symptom was noted in 18 children, 44.4% of whom required biologic treatment, compared to only 6.5% of the remaining UC subpopulation: Cramer’s V = 0.4218, Yule’s Q = 0.8413. The prevalence of other clinical manifestations did not differ significantly between the UC subpopulations (i.e., study and control); therefore, these symptoms could not be considered as predictive of biologics.

Regarding laboratory test results at diagnosis of UC, only hypoalbuminemia significantly elevated the risk of subsequent biologic therapy, and other tested parameters resulted as insignificant. The statistical parameters of UC clinical symptoms and additional test results are summarized in Table 6.

**Figure 4:**
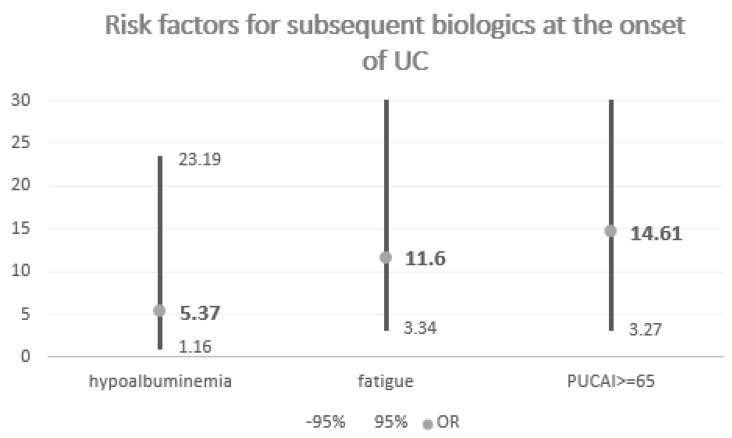
UC risk factors.

Apart from the above, the microbial antigens antibodies were also analyzed. No study participants, i.e., biologic therapy recipients, had positive ASCA IgG test results, while 14 control (not requiring biologics) children did (OR 0.2; 0.01–3.56; *p* = 0.13). Only 1 study child and 22 controls were IgA positive (OR 0.27; 0.03–2.15; *p* = 0.71). Double ASCA positivity was not found in the study group but was revealed in 12 control participants (OR 0.24; 0.01–4.25; *p* = 0.17). pANCA+ was noted at diagnosis in 1 patient that required biologics during the follow-up and in 10 children who did not require this treatment (OR 0.67; 0.08–5.67; *p* = 0.71). It can therefore be concluded that microbial antigens antibodies were not related to a higher risk of biologic therapy <18 y. The summary of significant parameters (OR) in UC analysis is presented in Figure 4.

### 3.3. IBD

Clinical and laboratory parameters that are meaningful and shared by both IBD forms (i.e., Crohn’s disease and ulcerative colitis) were jointly analyzed for the CD and UC populations so that a larger sample could be assessed. The mean age at IBD diagnosis was 152 months in the study group and 170 months in the control (*p* = 0.0067). IBD onset symptoms that turned out predictive of subsequent biologic therapy were fatigue and fever (Table 7). Other risk factors were hypoalbuminemia, hypoproteinemia, and CRP elevation.

Hypoproteinemia had the highest PPV for biologic therapy, but its sensitivity was extremely low. Compared to other risk factors, fatigue and CRP elevation showed relatively high sensitivities, i.e., 50–70% and 45–70%, respectively. The negative correlation between eosinophilic infiltrates in GI mucosa and subsequent biologics was close to statistical significance (OR = 0.43, CI 0.17–1.07, *p* = 0.069; Cramer’s V = 0.1223; Yule’s coefficient Q = −0.402, Z = −2.05, *p* = 0.04). Hence, it can be concluded that this type of infiltrate was related to a lower risk of subsequent biologic therapy.

No differences were observed between the study and control groups with respect to the remaining study parameters, i.e., underweight (37.75 vs. 29.2%, *p* = 0.25), overweight/obesity (20 vs. 23%, *p* = 0.74), growth impairment (G1, 26 vs. 19.6%, *p* = 0.29) and severe growth impairment (9.4 vs. 6%, *p* = 0.413), serum IgG elevation (14.3 vs. 8%, *p* = 0.2618), or total IgE elevation (approx. 23% in both groups). A statistical tendency for more frequent presentation of extraintestinal manifestations of IBD at diagnosis in patients ultimately treated with biologic therapy was noted (14 vs. 3.8%, *p* = 0.07; OR = 0.278, CI 0.06–1.22, *p* = 0.09)

## 4. Discussion

The definition and validation of predictors of poor outcome (POPO) in pediatric IBD have recently been extensively explored. Attempts have been made to develop evidence-based prognostic algorithms and therapies tailored to disease severity at presentation.

The 2020 ECCO-ESPGHAN guidelines on the medical management of pediatric Crohn’s disease [11] use POPO to select patients who are at low-, moderate- and high-risk of poor outcome and to recommend the respective first-line therapies for these groups, including exclusive enteral nutrition, steroids, anti-TNF, or surgery. Inclusion into a particular risk group is based on disease behavior, its extension, and additional risk factors, such as growth delay, perianal disease, and failure to induce remission after 12 weeks of induction therapy.

In our study, we analyzed selected clinical and laboratory parameters of children with IBD onset in order to identify potential risk factors for starting biologic therapy before the age of 18 years. Medical records for the years 2009–2019 were revised; at that time, risk stratification and patient qualification for biologics were not clearly defined by the ECCO-ESPGHAN guidelines. IBD management followed a step-up strategy; patients included in this study had unfavorable disease courses as evidenced by resistance to exclusive enteral nutrition or steroid resistance/steroid dependence otherwise referred to as unsuccessful standard PIBD care. The adoption of biologics was therefore chosen as the primary clinical endpoint.

Our observations are partially consistent with the literature (including the 2020 ECCO-ESPGHAN guidelines), where inflammatory behavior is associated with a low risk of poor outcome compared to other disease types. Complicated behavior has been found to correlate with several endpoints. PIBD-Ahead, a systematic review aimed at predicting outcomes in pediatric Crohn’s disease, concluded that B2/B3 disease predicted IBD-related surgery [20]. One of three studies that examined the association between PCDAI at diagnosis and subsequent treatment reported a correlation between PCDAI and the need for immunomodulators by one year. The Porto Group GROWTH Study revealed that stricturing disease at baseline and PCDAI > 10 at week 12 were key predictors for early surgery. Elevated PCDAI at week 12 also had an increased risk of surgery at follow-up [21].

Our analysis demonstrated that high PCDAI at diagnosis and complicated disease were related to the more frequent adoption of biologics. Biologic therapy recipients had higher PCDAI scores at baseline compared to non-recipients (47.63 vs. 41.59, *p* = 0.0441). Children with complicated disease at diagnosis had an almost twofold higher risk of subsequent biologic therapy (RR 1.932, 1.164–3.121, *p* = 0.0169). Perianal disease is also considered a high-risk factor for poor outcomes. In our study, perianal lesions strongly increased the likelihood of subsequent adoption of biologic therapy (OR 3.7; 1.204–11.68; *p* = 0.0356). Of course, based on the 2020 guidelines for pediatric Crohn’s disease management, complicated disease type and perianal disease refractory to local/antibiotic therapies has been an established and independent criterion for upfront biologic therapy. Therefore, it should be emphasized that this observation is made based on a retrospective cohort study.

According to the 2020 ECCO-ESPGHAN Guideline Update, extensive Crohn’s disease (i.e., affecting proximal and terminal ileum and the colon) is also among the risk factors for an unfavorable course and resultant biologics. In our study, L1 as well as the coexistence of L4a and L4b involvement were found to be predictors of subsequent biologic therapy.

In 2021, another consensus was reached regarding possible predictors of disease course in pediatric UC [22]. It was suggested that a PUCAI score ≥ 65 at diagnosis predicted colectomy and so did disease extent, low hemoglobin/hematocrit, and WBC elevation. Similarly, the authors of the PROTECT study concluded that low baseline clinical severity, high baseline hemoglobin, and week 4 clinical remission were associated with corticoid-free remission at week 52 [23]. We observed that higher disease activity at IBD diagnosis correlated with the risk for biologics before the age of 18. In our study, hypoalbuminemia at diagnosis was more prevalent in subsequent recipients of biologic therapy; the authors of the abovementioned consensus concluded that hypoalbuminemia predicted acute severe colitis but not colectomy. Age at diagnosis turned out to be a risk factor for malignancy but not for colectomy, disease extension over time, acute severe colitis, or medication intensification. Our analysis did not reveal any correlation between age at diagnosis and risk for biologics before the age of 18.

A large number of studies have focused on the presence of microbial antigens antibodies as potential prognostic markers for aggressive disease. A majority of researchers have concluded that ASCA positivity is associated with unfavorable events. In 2016, the IBD Porto Group of ESPGHAN presented the results of a multicenter retrospective longitudinal study on the serological profile of 406 children with colonic IBD (CC—Crohn’s Colitis, UC—Ulcerative Colitis, and IBDU—inflammatory bowel disease unclassified) [24]. UC children with pANCA+/ASCA- had more often moderate/severe disease at diagnosis and a higher risk for calcineurin inhibitors, biologics, or colectomy. In Crohn’s Colitis, double positivity for ASCA was associated with an aggressive disease course and, marginally, the need for biologics, but the pANCA-ASCA+ profile was of no significance. The systematic review of the Pediatric Inflammatory Bowel Disease Ahead Program [20] concluded that ASCA positivity correlates with a higher risk of IBD-related surgery; however, the authors emphasized that the conclusions of the reviewed papers were not consistent. Aloi et al. concluded that pediatric stricturing Crohn’s disease was associated with ASCA positivity [25]. Similar conclusions were reached by Kugathasan et al. [26].

We did not find any correlation between ASCA and pANCA and the adoption of biologic therapy. ASCA positivity was more frequent in Crohn’s disease controls (i.e., non-recipients). It seems hard to account for inconsistencies between our results and those reported by other authors. Although stricturing disease, surgical management, as well as ultimate adoption of biologics can be considered characteristic of unfavorable clinical course, these are ultimately different endpoints and can prove different correlations. Another possible explanation could be differences between study populations; unlike Liron Birimberg-Schwartz et al. in the ESPGHAN Report [24], we did not include patients with unclassified inflammatory bowel disease. It is also known that ASCA titres tend to vary over time [27,28] and differ depending on the patient’s age at IBD diagnosis [1].

Apart from the lack of correlation between ASCA serology and subsequent biologic treatment, we also observed that positive family history for IBD was more frequently noted in CD and UC non-recipients. On one hand, young age at diagnosis probably evidences high genetic load [29] and could be seen as a risk factor for unfavorable prognosis. On the other hand, a history of IBD in a close relative favors diagnosis at an earlier stage of the disease, which can influence the first years of patient care. A limitation of our study was the cut-off age of 18 and so we cannot discuss the clinical courses of our patients during longer follow-ups.

Some researchers have also considered the effect of overweight/obesity on IBD course. Although weight loss/underweight are among the symptoms of Crohn’s disease, it has been suggested that overweight/obesity, increasingly common among patients with IBD, might aggravate the clinical course due to the interplay between proinflammatory states of obesity and IBD-related inflammation [30,31]. Bearing this in mind, we examined the potential impact of overweight/obesity on the need for biological therapy; no correlation was revealed, although it should be emphasized that only 20 (9.5%) out of 231 IBD participants were overweight/obese.

## 5. Conclusions

Summing up, in Crohn’s disease sufferers, perianal disease, complicated disease type, ileocecal disease, hypoproteinemia, high PCDAI at diagnosis, elevated total serum IgA, and, marginally, anemia and L4a and L4b coexistence, turned out to be predictors of biologic therapy adoption before the age of 18 years. The odds ratio was the highest for elevated serum IgA, perianal disease, and complicated disease type. In UC, the risk factors were fatigue, hypoalbuminemia, high PUCAI at diagnosis, and extraintestinal manifestations other than primary sclerosing cholangitis. Fatigue and hypoalbuminemia were the most important. Generally, risk factors for biologic treatment in IBD (UC and CD together) were fever and fatigue at diagnosis, hypoalbuminemia, hypoproteinemia, and elevated CRP; hypoalbuminemia was the most significant predictor.

We believe that our observations regarding clinical manifestations of IBD at diagnosis and the effect thereof on the subsequent adoption of biologic therapy are the most interesting aspect of this study. It seems that children with severe IBD who subsequently require biologic therapy more frequently present with symptoms and signs of systemic inflammation, i.e., fatigue, fever, and CRP elevation. Interestingly enough, the proportions of hypoalbuminemia were comparable in UC (15%) and CD (19%). However, correlation with subsequent adoption of biologics was only noted in UC participants. These findings might indicate a need for a larger sample; a combined analysis of both UC and CD demonstrated the statistical significance of this parameter (OR = 2.69, *p* = 0.0113). It should be emphasized that, while PCDAI provides more detailed characteristics of the disease as it comprises albumin level, ESR, fever, and extraintestinal manifestations, PUCAI does not include an assessment of these features. Nevertheless, it seems that laboratory parameters, body temperature, or extraintestinal manifestations at diagnosis might have significant prognostic value also in UC.

Our findings in general are consistent with those reported by other authors. We hope they might contribute to the assessment of clinical presentation in IBD and the prognosis of its clinical course.

## Figures and Tables

**Table 1:** Paris classification. In brackets number of patients in each subgroup (presenting with each disease location/behavior) is given.

Location (Number of Patients)	L1 (64)	L2 (19)	L3 (36)
Of which biology demanding	40.63%	26.3%	22.22%
upper GI involvement	L4a (43)	L4b (14)	L4aL4b (6)
biology demanding	30.23%	35.71%	66.67%
Disease behavior	B1 (85)	B complicated (B2/B3) (34)	
biology demanding	25.88%	50%	
Perianal disease	Perianal disease present (15)	No perianal disease (104)	
biology demanding	60%	28.8%	
Linear growth impairment	G0 (90)	G1 (29)	G2 (8)
biology demanding	31.11%	37.93%	50%

**Table 2:** Paris classification-statistics.

Parameter (Number of Patients)	OR (95% CI)	RR (95% CI)	Sensitivity (95% CI)	Specificity (95% CI)	PPV (95% CI)	NPV (95% CI)	*p*
L1 (64)	2.21 1.004–4.97	1.72 1.002–3.04	0.6667 0.5098–0.7937	0.5250 0.4170–0.6308	0.4063 0.2946–0.5285	0.7636 0.6365–0.8563	0.0534
L4a (43)	0.83 0.37–1.88	0.88 0.50–1.49	0.3333 0.2063–0.4902	0.625 0.5155–0.7231	0.3023 0.186–0.4511	0.6579 0.5460–0.7546	0.6896
L4b (14)	1.16 0.41–3.77	1.10 0.49–2.07	0.1282 0.056–0.2671	0.8875 0.7998–0.9397	0.3571 0.1634–0.6124	0.6762 0.5818–0.7581	0.7710
L4aL4b (6)	4.457 0.99–23.95	2.15 0.93–3.41	0.1026 0.0406–0.2358	0.975 0.9134–0.9956	0.6667 0.3–0.9408	0.6903 0.5999–0.7681	0.0891
B complicated (34)	2.86 1.28–6.54	1.93 1.16–3.12	0.4359 0.293–0.5902	0.7875 0.6858–0.8629	0.5 0.3407–0.6593	0.7412 0.6391–0.8224	0.0169
p+ (15)	3.7 1.2–11.68	2.08 1.16–3.27	0.2308 0.1265–0.3834	0.925 0.8459–0.9652	0.6 0.3575–0.8018	0.7115 0.6182–0.7898	0.0356
Linear growth impairment (29)	1.35 0.57–3.22	1.219 0.68–2.05	0.2821 0.1654–0.4378	0.775 0.6721–0.8527	0.3793 0.2269–0.56	0.6889 0.5872–0.7751	0.5034
G2 (8)	2.17 0.59–7.76	1.59 0.66–2.79	0.1026 0.04–0.235	0.95 0.8784–0.9804	0.5 0.2152–0.7848	0.6847 0.5933–0.7637	0.4359

**Table 3:** CD symptoms. In brackets the number of patients presenting with each symptom is given.

	OR 95% CI	RR 95% CI	Sensitivity 95% CI	Specificity 95% CI	PPV 95% CI	NPV 95% CI	*p*
Fever (22)	2.46 0.94–6.45	1.73 0.94–2.79	0.2821 0.1654–0.4378	0.8625 0.7703–0.9215	0.5 0.3072–0.6928	0.7113 0.6145–0.7921	0.0777
Fatigue (74)	1.22 0.54–2.78	1.14 0.67–2.02	0.6579 0.4989–0.7879	0.3875 0.2882–0.4971	0.3378 0.2405–0.4512	0.7045 0.5578–0.8184	0.6874
Aphthous stomatitis (4)	6.58 0.94–86.36	2.39 0.93–3.64	0.07692 0.0265–0.2032	0.9875 0.9325–0.9994	0.75 0.3006–0.9872	0.6876 0.5973–0.7645	0.1024
EIM (7)	0.33 0.03–2.14	0.42 0.075–1.58	0.02564 0.00132–0.1318	0.9250 0.8459–0.9652	0.1429 0.0073–0.5131	0.6607 0.5690–0.7418	0.4240
Weight loss (61)	0.86 0.39–1.86	0.9 0.54–1.51	0.4872 0.3387–0.638	0.475 0.3692–0.583	0.3115 0.2094–0.4359	0.6552 0.5267–0.7644	0.8453
Underweight (44)	1.27 0.59–2.82	1.17 0.69–1.94	0.4103 0.2708–0.5658	0.6456 0.5356–0.7420	0.3636 0.2378–0.5113	0.6892 0.5766–0.7831	0.6860
Overweight /obesity (7)	0.81 0.156–4.1	0.87 0.24–2.08	0.0513 0.0091–0.1689	0.9375 0.8619–0.9730	0.2857 0.0508–0.6411	0.6696 0.5782–0.7499	>0.999
Rectal bleeding (37)	0.56 0.24–1.29	0.67 0.35–1.2	0.2308 0.1265–0.3834	0.65 0.5408–0.7455	0.2432 0.1336–0.4012	0.6341 0.5261–0.7302	0.2117
Diarrhea (77)	0.5 0.23–1.14	0.64 0.39–1.06	0.5385 0.3857–0.6843	0.3 0.2106–0.4077	0.2727 0.1858–0.3812	0.5714 0.4221–0.7088	0.1032
Abdominal pain (79)	0.86 0.38–1.92	0.9 0.54–1.56	0.641 0.4842–0.7726	0.325 0.2324–0.4336	0.3165 0.2245–0.4255	0.65 0.4951–0.7787	0.8365

**Table 4:** CD laboratory tests results. In brackets the number of patients presenting with each result is given.

	OR 95% CI	RR 95% CI	Sensitivity 95% CI	Specificity 95% CI	PPV 95% CI	NPV 95% CI	*p*
Hypoproteinemia (3)	1.83 to infinity	3.22 1.37–16.91	0.07692 0.0265–0.2032	1.0 0.9542–1	1.0 0.4385–1	0.6897 0.60–0.7666	0.0334
Elevated serum IgA (9)	4.67 1.23–17.58	2.22 1.12–3.45	0.1538 0.0725–0.2973	0.9625 0.8955–0.9898	0.6667 0.3542–0.8794	0.7 0.6088–0.7777	0.0572
Anemia (32)	2.316 0.99–5.34	1.69 1.01–2.75	0.3846 0.2489–0.541	0.7875 0.6858–0.8629	0.4688 0.3087–0.6355	0.7241 0.6223–0.8071	0.0768
Hypoalbuminemia (21)	2.16 0.83–5.81	1.61 0.89–2.63	0.2564 0.1457–0.4108	0.8625 0.7703–0.9215	0.4762 0.2834–0.6763	0.7041 0.6074–0.7854	0.1285
CRP (79)	1.447 0.62–3.2	1. 29 0.74–2.35	0.7179 0.5622–0.8346	0.3625 0.2657–0.4719	0.3544 0.258–0.4644	0.725 0.5717–0.8389	0.4160
Elevated serum IgG (8)	2.17 0.59–7.76	1.59 0.66–2.79	0.1026 0.0406–0.2358	0.95 0.8784–0.9804	0.5 0.2152–0.7848	0.6847 0.5933–0.7637	0.4359
thrombocytopenia (27)	1.23 0.57–2.76	1.15 0.67–1.9	0.3846 0.2489–0.541	0.6625 0.5536–0.7565	0.3751 0.2299–0.5083	0.6883 0.578–0.7807	0.6843
IgA ASCA (57)	0.95 0.43–2.07	0.96 0.57–1.62	0.4737 0.3248–0.6274	0.5125 0.4049–0.6189	0.3158 0.21–0.4448	0.6721 0.5472–0.7766	>0.9999
IgG ASCA (60)	0.9 0.42–1.95	0.93 0.56–1.56	0.472 0.3387–0.638	0.4875 0.3811–0.5951	0.3167 0.2131–0.4423	0.661 0.5337–0.7686	0.8466
ASCA double positivity (42)	1.23 0.57–2.76	1.15 0.67–1.9	0.3846 0.2489–0.541	0.6625 0.5536–0.7565	0.3571 0.2299–0.5083	0.6883 0.578–0.7807	0.6843
IgE elevation (42)	1.04 0.47–2.34	1.03 0.59–1.71	0.359 0.2274–0.5158	0.65 0.5408–0.7455	0.3333 0.21–0.4845	0.6753 0.5646–0.7694	>0.9999
IgE class ≥2 allergy (7)	0.81 0.16–4.1	0.87 0.24–2.08	0.0513 0.0091–0.1689	0.9375 0.8619–0.973	0.2857 0.0508–0.6411	0.6696 0.5782–0.7499	>0.9999
Eosinophilic infiltrates in GI mucosa (16)	0.43 0.13–1.47	0.54 0.18–1.3	0.079 0.0265–0.2032	0.8375 0.7416–0.9025	0.1875 0.0659–0.4301	0.65 0.5545–0.7356	0.2594

**Table 5:** UC—Paris classification parameters. In brackets the number of patients presenting with each parameter.

Parameter (Number of Patients)	OR 95% CI	RR 95% CI	Sensitivity 95% CI	Specificity 95% CI	PPV 95% CI	NPV 95% CI	*p*
E4 (46)	2.09 0.58–7.93	1.91 0.71–5.15	0.5714 0.3259–0.7862	0.6122 0.5133–0.7027	0.1739 0.0909–0.3072	0.9091 0.8155–0.9577	0.1913
Backwash ileitis (8)	2.56 0.46–14.12	2.17 0.58–8.05	0.25 0.0319–0.6509	0.8846 0.8071–0.9389	0.1429 0.0178–0.4281	0.9387 0.8715–0.9772	0.2673
Linear growth impairment (14)	1.29 0.33–5.16	1.26 0.39–4.09	0.15 0.032–0.3789	0.8804 0.7961–0.9388	0.2143 0.0466–0.508	0.8266 0.7379–0.8956	0.7091
Severe growth impairment (9)	2.17 0.4–11.66	1.9 0.5–7.23	0.2222 0.0281–0.6	0.8835 0.8053–0.9383	0.1429 0.0178–0.4281	0.9286 0.8584–0.9708	0.3578

**Table 6:** UC additional tests results. In brackets the number of patients presenting with the result.

Parameter (Number of Patients)	OR 95% CI	RR 95% CI	Sensitivity 95% CI	Specificity 95% CI	PPV 95% CI	NPV 95% CI	*p*
Fatigue (18)	11.6 3.34–40.27	6.89 2.72–17.47	0.4444 0.2153–0.6924	0.9355 0.8649–0.976	0.5714 0.2886–0.8234	0.8969 0.8186–0.9494	<0.0001
Fever (8)	0.99 0.11–8.7	0.99 0.15–6.64	0.125 0.0028–0.5265	0.8738 0.7939–0.931	0.0714 0.0016–0.3387	0.9278 0.8569–0.9704	0.992
Weight loss (33)	1.94 0.62–6.13	1.77 0.67–4.71	0.1818 0.0698–0.3546	0.8974 0.8079–0.9547	0.4286 0.1766–0.7114	0.7216 0.6214–0.8079	0.2503
Underweight (28)	1.23 0.35–4.29	1.2 0.41–3.53	0.1429 0.0403–0.3267	0.881 0.792–0.9414	0.2857 0.0839–0.581	0.7551 0.6579–0.8364	0.7414
Abdominal pain (56)	1.36 0.44–4.22	1.31 0.49–3.53	0.611 0.3862–0.7969	0.4737 0.3654–0.5845	0.2157 0.1249–0.3463	0.8372 0.7003–0.9188	0.5921
Rectal bleeding (94)	1.17 0.24–5.74	1.15 0.28–4.7	0.1277 0.0677–0.2124	0.8889 0.6529–0.9863	0.8571 0.5719–0.9822	0.1633 0.0963–0.2516	0.8459
Non-bloody diarrhea (19)	1.39 0.35–5.58	1.34 0.41 4.3	0.1579 0.0338–0.3958	0.8817 0.7982–0.9395	0.2143 0.0466–0.508	0.8367 0.7485–0.9037	0.6342
Overweight/ obesity (13)	1.32 0.26–6.69	1.27 0.32–5.05	0.1429 0.0178–0.4281	0.8878 0.808–0.9426	0.005 0.0012–0.0198	0.9962 0.9953–0.997	0.738
EIM (18)	0.39 0.05–3.23	0.43 0.06–3.07	0.5882 0.0013–0.2869	0.8632 0.7774–0.9250	0.0714 0.002–0.3387	0.8367 0.7485–0.9037	0.37
PSC or PSC/AIH (14)	0.2 0.01–3.56	0.23 0.01–3.62	0 0–0.2316	0.8571 0.7719–0.9196	0 0–0.2316	0.8571 0.7719–0.9196	0.1306
EIM other than PSC (4)	2.44 0.24–25.19	2.08 0.35–12.22	0.25 0.0056–0.8059	0.8796 0.803–0.9343	0.0714 0.0016–0.3387	0.9694 0.9131–0.9936	0.4414
Hypoalbumi nemia (14)	5.37 1.16–23.19	3.89 1.52–9.95	0.3571 0.1634–0.6124	0.9082 0.8346–0.9509	0.3571 0.1634–0.6124	0.9082 0.8346–0.9509	0.0049
Elevated CRP (16)	1.78 0.44–7.26	1.64 0.51–5.23	0.1875 0.0405–0.456	0.8854 0.8042–0.9414	0.2143 0.0466–0.508	0.8673 0.7838–0.9274	0.4142
Hyperproteinemia (14)	0.85 0.17–4.19	0.87 0.21–3.56	0.1111 0.0137–0.35	0.8723 0.79–0.93	0.1429 0.0178–0.4282	0.8367 0.7485–0.903	0.85
Eosinophilic infiltrates in GI mucosa (13)	0.68 0.17–2.63	0.71 0.21–2.38	0.0967 0.0204–0.26	0.8642 0.77–0.93	0.2143 0.0466–0.508	0.7143 0.6142–0.801	0.58
Total IgG (13)	0.98 0.19–4.88	0.98 0.24–4.05	0.1176 0.0146–0.3644	0.8804 0.7961–0.9388	0.1538 0.0192–0.3644	0.8804 0.7961–0.9388	0.98

**Table 7:** IBD—predictors of biologics.

Parameter (Number of Patients)	OR 95% CI	RR 95% CI	Sensitivity 95% CI	Specificity 95% CI	PPV 95% CI	NPV 95% CI	*p*
Fever (53)	2.59 1.15–5.79	1.95 1.16–3.27	0.4 0.2266–0.5939	0.795 0.7323–0.8487	0.2264 0.1228–0.362	0.8983 0.844–0.9386	0.018
Fatigue (92)	3.47 1.82–6.62	2.59 1.56–4.26	0.3586 0.2613–0.4654	0.8613 0.7919–0.9143	0.6346 0.4896–0.7638	0.6667 0.592–0.7356	0.0001
Hypoalbuminemia (34)	3.28 1.53–7.05	2.28 1.42–3.66	0.4418 0.2719–0.6211	0.8061 0.7437–0.859	0.283 0.1679–0.4235	0.8927 0.8375–0.9341	0.0016
Hypoproteinemia (5)	14.23 1.58–132.25	3.69 2.23–6.11	0.07547 0.0297–0.1821	0.9944 0.9691–0.9999	0.8 0.3136–0.9722	0.7835 0.725–0.8349	0.0105
↑CRP (95)	2.51 1.34–4.69	2.02 1.26–3.25	0.5849 0.4509–0.7074	0.6404 0.5677–0.7073	0.3263 0.2404–0.4257	0.8382 0.7672–0.890	0.0042

## Data Availability

The Excel file with the original data has been sent to MDPI as a supplementary file.

## Supplementary Materials

The following are available online at https://www.mdpi.com/article/10.3390/children9101558/s1, Table S1: original data of analyzed patients in excel file.
